# Jumping to male-dominated occupations: A novel way to reduce the gender wage gap for Chinese women

**DOI:** 10.1016/j.heliyon.2023.e14198

**Published:** 2023-03-01

**Authors:** Wei Bai, Zhongtao Yue, Tao Zhou

**Affiliations:** CompleX Lab, University of Electronic Science and Technology of China, Chengdu 611731, China

**Keywords:** Gender wage gap, Occupational segregation, Gender inequality

## Abstract

Occupational segregation is widely considered as one major reason leading to the gender discrimination in labor market. Using large-scale Chinese re-sume data of online job seekers, we uncover an interesting phenomenon that occupations with higher proportion of men have smaller gender wage gap measured by the female-male ratio on wage. We further show that the sever-ity of occupational segregation in China is low both overall and regionally, and the inter-occupational discrimination is much smaller than the intra-occupational discrimination. That is to say, Chinese women do not face large barriers when changing their occupations. Accordingly, we suggest Chinese women a new way to narrow the gender wage gap: to join male-dominated occupations. Meanwhile, it is worth noticing that although the gender wage gap is smaller in male-dominated occupations, it does not mean that the gender discrimination is smaller there.

## Highlights

• Chinese women can reduce the gender wage gap by jumping to male-dominated occupations.
• The barrier for Chinese women to change occupations is lower than the Western.
• Inter-occupational discrimination is much smaller than intra-occupational discrimination.

## 1 Introduction

As one of the 17 Sustainable Development Goals, reducing gender in-equality is a major policy concern around the world [1]. The gender wage gap is a prominent part of gender inequality. We use the ratio of females' wage to males’ (female-male ratio, $r_{fm}$) as the primary measure of the gender wage gap. Globally, $r_{fm}\approx 0.76$ [2]. Zhang et al. [3] showed that from 1988 to 2004, $r_{fm}$ in China decreased from 0.863 to 0.762. Although there are fluctuations in some years, it is very clear that the gender wage gap keeps widening over time.

The gender wage gap is often closely related to occupational gender segregation (occupational segregation for short) [4,5], which refers to the fact that workers in the labor market are assigned to different occupational categories due to gender differences, observed as the concentration of most female labor force in some “feminine” occupations with low wage and low prestige. Occupational segregation is usually considered as a main way of gender dis-crimination and a major cause of the gender wage gap. Among millennials, occupational segregation accounts for 28% of the gender wage gap [6]. Meanwhile, He et al. [7] show that the impact of occupational segregation on gender wage gap increases with the marketization in prefectural level. In terms of professions, male and female students have different educational processes and outcomes even in the same fields. Although STEM occupations are often high-paying, women in them tend to be concentrated in lower-wageing ones [8]. Even when women get the highest paying jobs in computer science and engineering, they still earn less than their male counterparts. In different regions of the United States, the wage of female doctors is generally lower than that of male [9]. In Brazilian tourism industry, women are valued less than men even when they have the same occupational characteristics [10]. In finance, although American male and female MBA graduates earn almost the same at the start of their careers, the ratio of females' logarithmic annual wage to males’ has been as low as 0.625 after 10–16 years [11]. Gender differentiation has also appeared in different sectors in China: the gender wage gap within the political sector is gradually disappearing, while outside is widening [12]. Yang et al. [13] notice that Chinese male job seekers have remarkably higher salary expectation than females. In the American public sector, occupational segregation is still the main cause of wage disparity [14].

The impact of occupational segregation on the gender wage gap also varies by country and generation. Todd et al. [15] find that occupational segregation increases in Australia from 1995 to 2011. However, Busch [16] finds that women's earnings in male-dominated occupations increases in Germany between 1992 and 2015. By using data from the European Structure of Earnings Survey (2010), Boll et al. [17] show that sectoral isolation has 0%–15% explanatory power in salary decomposition. Laine [18] analyzes the impact of different types of occupational segregation in Finland and finds that the explanatory power of corporate and job segregation drops from 23.6% to 19.1% between 1995 and 2004. Using data from the 1988–1992 Chinese Urban Household Survey, Ng [19] finds that the total explanatory power of occupational segregation is 20%–40%, after controlling the influence of education, work experience, province, industry, occupation, and company ownership.

Recently, the discussion of the factors affecting occupational segregation is controversial. Blau et al. [20] find that the largest decrease in occupational segregation occurs in American college graduates from 1970 to 2009, which is related to the improvement of education. However, Borrowman et al. [21] show that rising education levels tend to increase rather than decrease segregation in developing countries between 1980 and 2011. In addition, the demand side of the labor market also creates occupational segregation. For example, differences in the operating mechanisms and hiring restrictions of different ownership sectors can lead to different preferences of women and men in employment. The private sector may value individual productivity more than the state sector, leading to a preference for male employees, while the public sector is more protective of women. Using data from the Chinese Household Income Project survey (CHIP2002 and CHIP2013) with Brown decomposition model, Ma [22] finds that the discrimination against female workers in a given ownership sector is becoming more serious and is the main factor causing the gender wage gap expansion in urban China from 2002 to 2013. Maurer-Fazio et al. [23] find that the gender wage gap is greatest within joint ventures, followed by the collective sector and then state-owned enterprises. Blau et al. [24] analyze the influence of gender on career promotion and find that the probability of female promotion is low, also contributing to the emergence of occupational segregation.

Unfortunately, occupational and sectoral segregation has increased over time in many countries [21]. According to the 2021 Global Gender Gap Report, it will take 145.5 years to achieve gender equality in politics, especially as leadership positions continue to be underserved by women, who account for only 27% of all managerial positions [25]. In the past, suggestions for reducing occupational segregation have focused on the demand side, relying on the policy implementation. Zhang et al. [3] find that the narrowing of the gender wage gap in China in the 1990s is caused by institutional changes, such as the enactment of the Labor Law in 1994, which protects women's rights. The rights of paid maternity leave is conducive to reducing women's reproductive pressure and facilitating their career development [26]. In the United States, Blau and Kahn [27] show that career advancement and de-unionization have helped raise women's relative wages. However, relying only on policy changes is relatively passive and uncontrollable for female workers, so recent studies have also looked at the supply side. Using event history analysis, Li [28] finds that occupation mobility has a negative effect on earnings returns, especially for women. That is, it is better for women to reduce occupational mobility and maintain a more stable job. Using data from the National Longitudinal Survey of Youth 1979 (NLSY79), Pearlman [29] finds that women with a bachelor's degree employed in highly male-dominated occupations can use voluntary inter-firm mobility to narrow the gender wage gap. The evidence from Poland shows that the wage discrimination in gender balanced occupations is the smallest [30].

Up to now, most related studies rely on questionnaire survey [31] or experimental data [32], both of which are based on small samples. This work explores the relationship between occupational segregation and the gender wage gap at occupational granularity by using large-scale samples. Our aim is to make practical suggestions to women about how to reduce the gen-der wage gap. The remainder of this paper is organized as follows. Section 2 presents the relationship between gender wage gaps and levels of occupational segregation in different occupations, as well as the overall severity of Chinese occupational segregation. Section 3 analyzes the severities of discrimination in different occupations by decomposing wages into those determined by human endowments and those caused by discrimination. Section 4 further decomposes the gender wage gaps into intra- and inter-occupational differences. Finally, Section 5 briefs the conclusions along with some discussions.

## 2 Gender wage gap and occupational segregation

We use resume data of 10,318,484 job seekers crawled from various online recruitment websites from 2014 to 2015. The data includes basic personal in-formation, educational experience, work experience and expected occupation and position. Among them, gender, age, previous wage and occupation from work experience are mainly utilized. A job seeker is accepted for further analysis if the information about gender, age, last year salary and occupation are all presented, and the age is at least 16 years old. After data screening, 3,266,272 job seekers are accepted, including 1,957,747 for males and 1,308,525 for females.

**Fig. 1:**
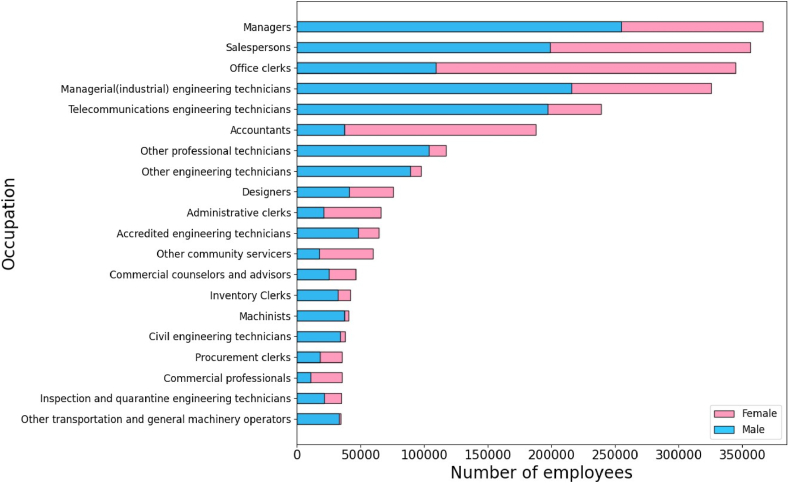
The number of employees of the top-20 occupations.

In the following analysis, we mainly focus on the top-20 occupations with the largest number of employees. The total number of employees in those 20 occupations is 2,607,503, accounting for 79.83% of the entire samples, as detailed in Fig. 1.

**Fig. 2:**
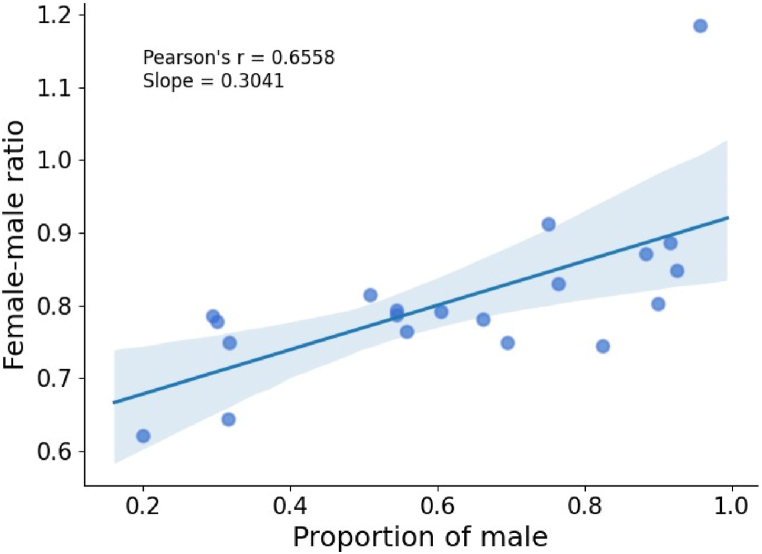
The relationship between proportions of male employees and female-male ratios in the top-20 occupations. The line is the linear fit of the scatter plot, and the blue shaded area is the 95% confidence interval. (For interpretation of the references to color in this figure legend, the reader is referred to the Web version of this article.)

In Fig. 2, we present the relationship between gender wage gaps and severities of occupational segregation (measured by proportions of male employees) in the top-20 occupations. Though the gender wage gaps exist (i.e., $r_{fm}<1$) in almost all occupations, it is observed that male-dominated occupations (such as engineering and professional technicians) have relatively smaller $r_{fm}$, while accountants, office clerks and other occupations that are generally perceived as more feminine have relatively larger $r_{fm}$. The result suggests that whether the occupation is dominated by men is significantly related to the gender wage gap (the Pearson correlation coefficient reaches 0.6558, with *p*-value*<*0.01 according to the Student's *t*-test). To some extent, the observed correlation indicates that although male-dominated occupations may have higher entry barriers for women, they exhibit narrower the gender wage gap than female-dominated ones.

We calculate one of the most classic occupational segregation indices, the Duncan index [33], which is a measure of what percentage of workers of another gender would have to change jobs in order to achieve an equal distribution of genders across occupations, when workers of one gender stayed in their current jobs. The Duncan index ranges from 0 to 1: if all occupations are completely dominated by one gender, it equals 1, while if proportions of male employees in all occupations are the same, it equals 0. The mathematical formula of the Duncan index is shown in Equation (1):

(1) $$D=\frac{1}{2}\sum_{j=1}^{n}\left|\left(F_{j}/F\right)-\left(M_{j}/M\right)\right|\text{,}$$

where $F_{j}$ and $M_{j}$ are the number of female and male employees in occupation j respectively, F and M are the number of all female and male employees, and *n* is the number of considered occupations.

**Fig. 3:**
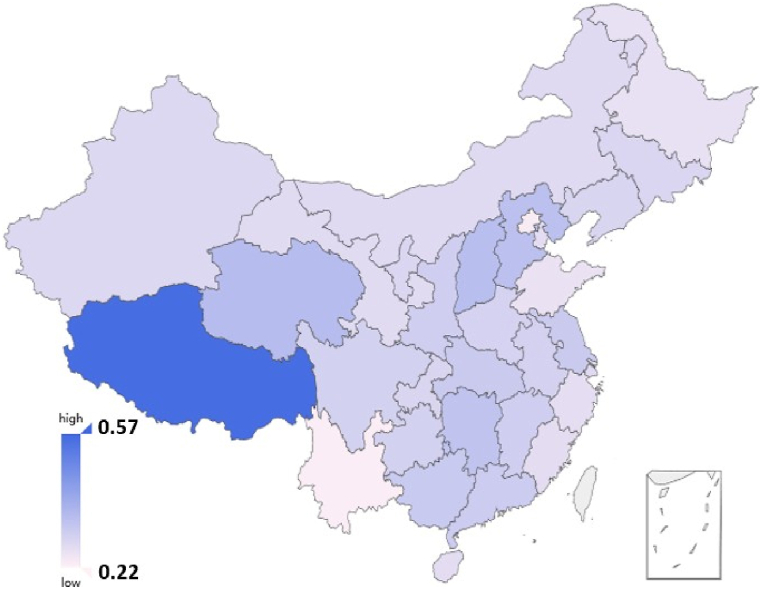
The Duncan indices for Chinese provinces.

The Duncan index for the current data set is 0.398, which is remarkably lower than those of developed countries in the world (for example, the Duncan index in the United States has decreased from about 0.60 to 0.47 in the recent 60 years [34], and in Finland and Sweden, the Duncan index is still more than 0.40 in 2020 after a long decline [35]). At the same time, the Duncan index based on the 2010 Population Census of the People's Republic of China is 0.243, even lower than the resume data. Fig. 3 shows the Duncan indices for different provinces with lighter color corresponding to less segregation. One can observe that, except for Tibet, severities of occupational segregation for different provinces are similar and low. As Tibet is an outlier, it is removed from the later statistics.

**Fig. 4:**
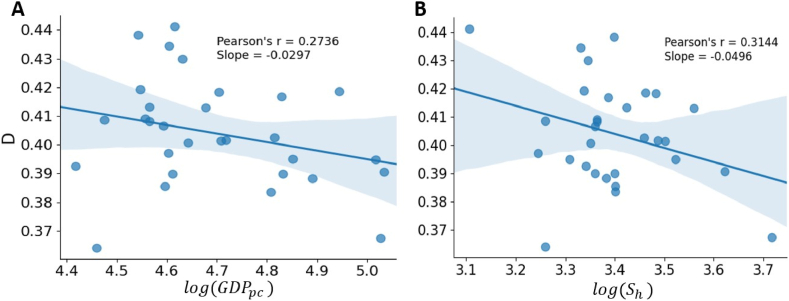
The relationship between *D* and *GDP*_*pc*_ (A), and the relationship between *D* and *S*_*h*_ (B) at the provincial level. The lines are the linear fits of the scatter plots, and the blue shaded areas are the 95% confidence intervals. (For interpretation of the references to color in this figure legend, the reader is referred to the Web version of this article.)

Fig. 4 shows the relationships between the Duncan index and per capita GDP (*GDP*_*pc*_) and the number of college students per 10^5^ people (*S*_*h*_). As the majority of updatings in the resume data happened in 2015, the relevant statistical data at provincial level are taken from the China Statistical Year-book 2016, which presents statistics of social and economic status in 2015. It can be seen that the Duncan index is significantly correlated with the level of economy and education (*p*-values are all less than 0.01 according to the Student's *t*-test). The above result suggests that the improvement of education and economy is helpful in reducing occupational segregation, however, the change of *D* is not remarkable.

## 3 Inter-occupational gender discrimination

Wage decomposition methods are used to analyze differences between variable distributions in different groups, where samples are usually grouped by gender or race. Therefore, to better understand the impacts of discrimi-nation on the gender wage gap, we apply two wage decomposition methods in this work. This section presents the first one, called Brown decomposition, which takes the occupational segregation factor into the wage decomposition, and the gender wage gap is then decomposed into intra-occupation and inter-occupation differences [36]. The next section applies the so-called Blinder- Oaxaca (BO) decomposition, which decomposition establishes a regression model for all considered factors that may affect wage, and then calculates regression coefficients and the gender gap that can be explained by those considered factors [37,38].

First, we study the impact of occupation-related discrimination on the gender wage gap, we implement Brown decomposition on the resume data. Specifically, the gender wage gap is decomposed into differences within and between occupations, and gender occupational distribution is estimated from the perspective of job acquisition, thus separating out the inequality caused by gender-specific occupational entry barriers. In this paper, considering the distribution of individual occupations as an endogenous variable, we use the two-stage process and multiple choice model to estimate the entry probability of female (or male) in “barrier-free” career choice. Finally, the Mincer wage equation is used to estimate the wage function of men and women in each occupation. Brown decomposition emphasizes the impact of occupational segregation on wage gaps and constructs a counterfactual framework where women face the same occupational structure as men. The mathematical formula of Brown decomposition is formulated in Equation (2):

$${\bar{S}}_{M}-{\bar{S}}_{F}=\underset{PD}{\underbrace{\sum_{j}p_{j}^{F}\left({\bar{X}}_{j}^{M}-{\bar{X}}_{j}^{F}\right)\beta_{j}^{M}}}+\underset{WD}{\underbrace{\sum_{j}p_{j}^{F}{\bar{X}}_{j}^{F}\left({\bar{\beta }}_{j}^{M}-{\bar{\beta }}_{j}^{F}\right)}}$$

(2) $$+\underset{QD}{\underbrace{\sum_{j}{\bar{S}}_{j}^{M}\left(p_{j}^{M}-{\tilde{p}}_{j}^{F}\right)}}+\underset{OD}{\underbrace{\sum_{j}{\bar{S}}_{j}^{M}\left({\tilde{p}}_{j}^{F}-p_{j}^{F}\right)}}\text{,}$$

where S represents the average annual wage, X is the influencing factors of wage (i.e. the characteristic matrix), $p_{j}$ is the probability of an employee working in occupation j, M and F are short for male and female, and ${\tilde{p}}_{j}^{F}$ is the expected probability of $p_{j}^{F}$ if both occupational distributions of two genders are the same. PD and QD are the intra-occupational and inter-occupational gender differences of endowments, respectively. WD is intra-occupational gender wage discrimination, and OD accounts for the occupational segregation.

**Fig. 5:**
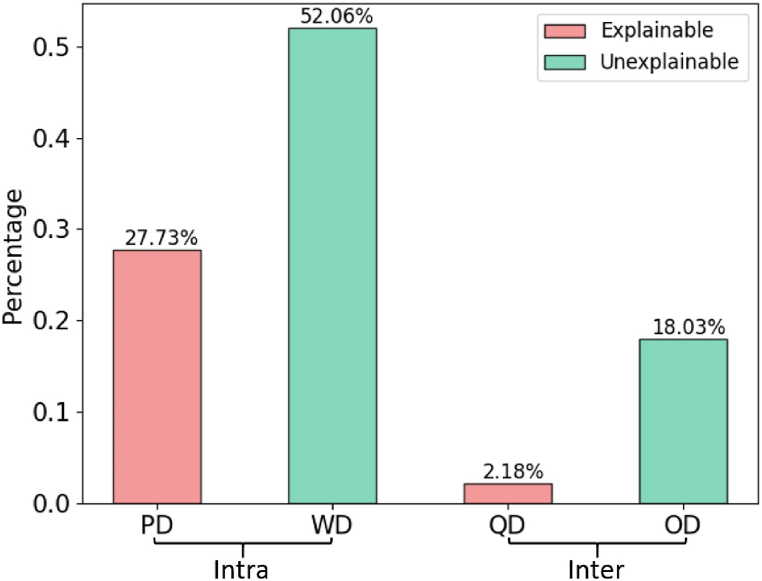
The result of Brown decomposition. PD and WD are intra-occupational differ-ences, and the latter two are inter-occupational differences. (For interpretation of the references to color in this figure legend, the reader is referred to the Web version of this article.)

The result of Brown decomposition is shown in Fig. 5, where WD ac-counts for the largest proportion (52.06%), and QD accounts for the smallest proportion (2.18%). It can be seen that the inter-occupational discrimination is low, which is in line with the fact that the Duncan index is also low. This indicates that occupations are not much segregated by gender, and thus women are relatively easy to jump to male-dominated occupations to avoid being trapped in occupations with very small $r_{fm}$.

## 4 Intra-occupational gender discrimination

According to the result of Brown decomposition, the intra-occupational gender discrimination is the main cause of the gender wage gap (79.79%). We then apply the BO decomposition for each occupation, which decomposes wage gaps between gender groups into the explainable part caused by differ-ences in individual characteristics, and the unexplainable part attributed to discrimination. Regression of the wage yields Equation (3):

$$S_{M}=\beta_{M}X_{M}+\varepsilon_{M}\text{,}$$

(3) $$S_{F}=\beta_{F}X_{F}+\varepsilon_{F}\text{,}$$

where β is the regression coefficient that captures the effects of human en-dowment on wage for male (or female), and *ε* is the error term. Then, the gap $1-r_{fm}$ can be expressed as Equation (4):

(4) $$1-r_{fm}\approx \left({\bar{X}}_{M}-{\bar{X}}_{F}\right)\beta_{M}+\left({\bar{\beta }}_{M}-{\bar{\beta }}_{F}\right){\bar{X}}_{F}\text{,}$$

where $\left({\bar{X}}_{M}-{\bar{X}}_{F}\right)\beta_{M}$ represents the gap coming from the differences in individual characteristics assuming that there is no gender discrimination (explainable part), and $\left({\bar{\beta }}_{M}-{\bar{\beta }}_{F}\right){\bar{X}}_{F}$ is the gap resulted from discrimination (unexplainable part).

In the following analysis, we concentrate on job seekers whose gender, age, seniority, degree, school, last year salary, marital status, profession, industry, occupation, expected city, living city and home city are all known (in total there are 753,616 such job seekers). In addition, the wage is taken logarithm, and variables except salary and dummy variables are treated centrally before decomposition.

The proportions of the explainable and unexplainable parts are formulated in Equation (5):

$$P_{e}=\frac{\left({\bar{X}}_{M}-{\bar{X}}_{F}\right)\beta_{M}}{\left({\bar{X}}_{M}-{\bar{X}}_{F}\right)\beta_{M}+\left({\bar{\beta }}_{M}-{\bar{\beta }}_{F}\right){\bar{X}}_{F}}\text{,}$$

(5) $$P_{u}=\frac{\left({\bar{\beta }}_{M}-{\bar{\beta }}_{F}\right){\bar{X}}_{F}}{\left({\bar{X}}_{M}-{\bar{X}}_{F}\right)\beta_{M}+\left({\bar{\beta }}_{M}-{\bar{\beta }}_{F}\right){\bar{X}}_{F}}\text{,}$$

**Fig. 6:**
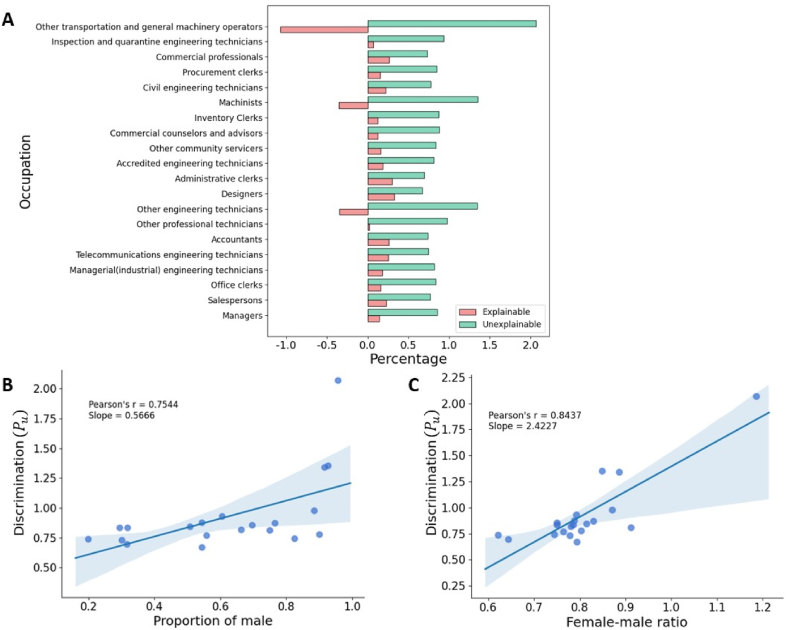
(A) The proportions of explainable (*P*_*e*_) and unexplainable (*P*_*u*_) parts in different occupations. (B) The relationship between the severities of occupational segregation and the proportions of the discrimination parts according to the BO decomposition. (C) The relationship between the gender wage gaps and the proportions of the discrimination parts according to the BO decomposition. The lines are the linear fits of the scatter plots, and the blue shaded areas are the 95% confidence intervals. (For interpretation of the references to color in this figure legend, the reader is referred to the Web version of this article.)

Obviously, $P_{e}+P_{u}=1$ and $P_{u}$ is usually considered as discrimination. Overall speaking, according to the BO decomposition, $P_{e}$ is only 18.53%, while $P_{u}$ = 81*.*47%. Notice that, if *P*_*e*_ *<* 0, for example $P_{e}=0.1$, it means female employees should earn 0*.*1*G* more than male employees according to their individual characteristics, where *G* denotes the gender wage gap. That is to say, the discrimination equals 1.1*G*, even larger than the observed gap. Fig. 6A shows the results of BO decomposition for the top-20 occupations, showing that the discrimination ($P_{u}$) of the gender wage gap is all relatively high, and even exceed 1 for a few occupations. Fig. 6B presents the relationship between the discrimination $P_{u}$ and the severities of occupational segregation, showing a strong and significant correlation (*p*-value*<*0.01 according to the Student's *t*-test). In addition, as shown in Fig. 6C, $P_{u}$ and $r_{fm}$ are also strongly correlated. In other words, although $r_{fm}$ in a male-dominated occupation may be smaller, the corresponding discrimination $P_{u}$ is very probably larger than a female-dominated occupation.

**Fig. 7:**
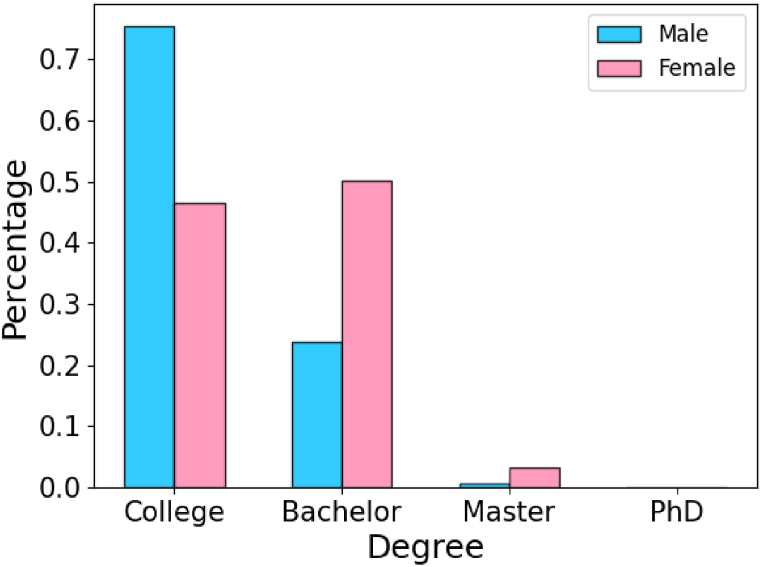
Distribution of the education experiences of male and female employees in “other transportation and general machinery operators”.

In the above analysis, the occupation “other transportation and general machinery operators” is an outlier. In this occupation, $r_{fm}=1.19$, the proportion of men is 96%, while $P_{u}>200\%$. Looking into the educati-on experiences of employees in this occupation (see Fig. 7), we find that more than 75% of men are junior college students, while more than half of women have a bachelor's degree or even above. This means that women's high wage is achieved through a better educational background. While it is a good strategy to narrow the gender wage gap by jumping to male-dominated occupations, women also need to pay more efforts than men, and in those less-gap occupations, the discrimination may be even larger (see Fig. 6C).

## 5 Conclusions and discussion

In this paper, using Chinese resume data from $∼3.3\times 10^{6}$ online job seekers, we study the severity of occupational segregation in China and its impact on the gender wage gap. Our results show that the gender wage gap in male-dominated occupations is relatively small, while the occupational segregation is not serious (indicated by a smaller Duncan index than many other countries) and the inter-occupational discrimination is low (according to the Brown decomposition). Therefore, to join male-dominated occupations is a feasible way to narrow the gender wage gap. However, we also show that the occupations with smaller gender wage gaps usually suffer even larger gender discrimination. That is to say, in those occupations, female employees ought to earn much more than male employees according to their individual characteristics. As all results come from large-scale natural data, we believe the reported phenomena are statistically solid [39,40]. At the same time, the results are limited by the features of data. Firstly, all samples are Chinese job seekers, so that the robustness of the results needs further validation by data from other countries. Secondly, the data are from 2014 to 2015, and thus newer data may bring novel insights, in particular, increasing remote jobs are created recently, which may benefit women who would like to work from home. Thirdly, the present data only covers occupations that could recruit employees online, while a considerable portion of occupations mainly recruit employees offline.

Occupational segregation mainly comes from the innate physiological characteristics and physiques and the resulting differences in acquired skills of men and women. Men are physically superior to women, while women have innate advantages in communication and language. In terms of indus-try distribution, men are concentrated in manufacturing, construction and telecommunication sectors, while the proportion of women in services such as education and medical care is relatively high. Whether in male-dominated or female-dominated occupations, women's wage and time spent are generally lower than men's [41,42]. Studies have also shown that the occupational segregation may be due to women's reluctance to choose skilled occupations [43].

Gender equality is a basic state policy of China, and Chinese government has made great efforts in reducing gender discrimination in the workplace, such as the promulgation of the *Law of the People's Republic of China on the Protection of Rights and Interests of Women*, which stipulates that women have equal rights with men in all aspects of political, economic, cultural, social and family life, and prohibits discrimination, abuse, abandonment and mutilation of women. The *Labor Law* also clearly states that women have equal employment rights with men, and emphasizes that the distribution of wages should follow the principle of *equal pay for equal work*.

In despite of considerable achievements in China, there is much room for improvement, especially in the protection of equal work opportunities and occupational treatment of women [44]. For example, the original intention of the paid maternity and parental leave is to reduce the family constraints on female career development, but the policy to some extent backfires [45]. For the demand side, as the costs of maternity and parental leave are al-most entirely borne by enterprises, women of childbearing ages are indeed out of favour by some private enterprises [46]. For the supply side, long work interruptions by maternity and parental leaves may go against career devel-opment [47]. Accordingly, the related policies could be improved by properly sharing costs of maternity and parental leave, such as the tax preference for enterprises with more than a threshold fraction of employees being mothers, and the cash subsidy for enterprises if their long-term hired employees bear children. In addition, the government should allow diverse maternity and parental leaves, especially encouraging a shorter leave followed by a long period of working from home. To directly reduce the discrimination on women of childbearing ages, the government may also provide male employees a parental leave that is similar to female employees, after building an effective mechanism to reduce the resulted costs from enterprises [48]. Meanwhile, it is necessary for the government to boost the supply of all-inclusive care services, so that women could choose between home and work from their own hearts, instead of being forced into family caring tasks. Only by freeing women from family responsibilities and unpaid caring work, can occupational segregation be essentially reduced.

To reduce the occupational segregation, known studies mainly consider the demand side. For example, in terms of political management of enter-prises, a gender quota system can be adopted to ensure a minimum proportion of female managers [49]. However, Shaikh et al. [50] show that social policies don't seem to affect intra-group gender distributions and thus ad-dress inequality in wage distribution. Our results provide a solution from the supply side of the labor market. In contemporary China, if women want to narrow the gender wage gap, they can jump to male-dominated occupations to obtain the same remuneration as men. And if women want to successfully join the male-dominated occupations, it is important to actively cultivate traditionally male-characteristic skills. It doesn't mean forcing women to do things they're not good at. Instead, women need to take the initiative to break occupational stereotypes. Nowadays, the training of occupation-related skills is usually not gender-specific. Taking engineering technicians as an example, women can receive relevant training and enter vocational and technical schools for systematic study. In fact, women can sometimes do better than men in engineering because they are more careful [51]. It is also important to choose an appropriate occupation. For example, knowledge-intensive industries which require skilled labor tend to be more gender-neutral and have smaller gender wage gap [52]. But at the same time, women should also be prepared for more severe discrimination. Indeed, a lower gender wage gap is statistically associated with higher gender discrimination. Before the arrival to true general equality, the improvement of personal endowment seems to be the only credible and critical way to break the wage limit.

## Author contribution statement

Wei Bai: Conceived and designed the experiments; Performed the experiments; Analyzed and interpreted the data; Contributed reagents, materials, analysis tools or data; Wrote the paper.

Zhongtao Yue: Contributed reagents, materials, analysis tools or data.

Tao Zhou: Conceived and designed the experiments; Analyzed and interpreted the data; Wrote the paper.

## Funding statement

This work was supported by National Natural Science Foundation of China [11975071], Ministry of Education of Humanities and Social Science Project [21JZD055].

## Data availability statement

The data that has been used is confidential.

## Declaration of interest’s statement

The authors declare no competing interests.
